# Pump–probe experimental methodology at the Linac Coherent Light Source^1^

**DOI:** 10.1107/S160057751900225X

**Published:** 2019-04-26

**Authors:** James M. Glownia, Karl Gumerlock, Henrik T. Lemke, Takahiro Sato, Diling Zhu, Matthieu Chollet

**Affiliations:** a Linac Coherent Light Source, SLAC National Accelerator Laboratory, 2575 Sand Hill Road, Menlo Park, CA 94025, USA; b SwissFEL, Paul Scherrer Institute, WBBA/022, 5232 Villigen PSI, Switzerland

**Keywords:** XFELs, X-ray free-electron lasers, ultrafast lasers, pump–probe experiments

## Abstract

Experimental methods that use free-electron laser (FEL) sources that can deliver short X-ray pulses below a 10 fs pulse duration and traditional optical lasers are ideal tools for pump–probe experiments. However, these new methods also come with a unique set of challenges, such as how to accurately determine temporal overlap between two sources at the femtosecond scale and how to correct for the pulse-to-pulse beam property fluctuations of the FEL light derived from the self-amplified spontaneous emission process. Over the past several years of performing pump–probe experiments at the Linac Coherent Light Source (LCLS), new methods and tools have been developed to improve the ways experimental timing is measured, monitored and scanned. The aim of this article is to present an overview of the most commonly used techniques at LCLS to perform pump–probe-type experiments.

## Abstract 2

Techniques developed at LCLS to make optical laser pump–X-ray probe experiments both robust and easy to implement at ultrafast X-ray free-electron lasers are presented.

## 1. Introduction   

The advent of free-electron laser based X-ray sources has unleashed a flurry of scientific investigations into the time-resolved evolution of atoms, molecules, bio-systems and solid-state systems. One of the most important methods used at these light sources for time-resolved studies is the pump–probe technique, where an optical laser pulse initiates dynamics that are later probed by an X-ray pulse. The X-ray probe measurement can employ a wide variety of methods including scattering, diffraction, emission, X-ray near-edge absorption, *etc.* to capture the dynamics (Wall *et al.*, 2018 ▸; Gray *et al.*, 2018 ▸; Teitelbaum *et al.*, 2018 ▸; Miller *et al.*, 2018 ▸; Zerdane *et al.*, 2018 ▸). In the evolution following the first time-resolved optical/X-ray studies at LCLS (Glownia *et al.*, 2010 ▸; Daranciang *et al.*, 2012 ▸; Lemke *et al.*, 2013 ▸), there have been many advances in how such experiments are performed (Seddon *et al.*, 2017 ▸). The experimental time resolution has been increased from 120 fs root mean square (RMS) to 5 fs RMS through the use of a Time Tool device that consists of an optical/X-ray cross correlator (Bionta *et al.*, 2011 ▸; Schorb *et al.*, 2012 ▸; Hartmann *et al.*, 2014 ▸; Medvedev *et al.*, 2013 ▸; Mecseki *et al.*, 2018 ▸), and the range of available optical wavelengths has expanded to cover from 200 nm to the THz. In the course of developing the sources and performing experiments, we discovered that the set of challenges in a FEL/optical pump–probe experiment is essentially universal to all experiments: synchronizing the optical lasers to the X-rays (Gumerlock *et al.*, 2014 ▸), finding spatial overlap between the optical laser and X-rays, finding temporal overlap, characterizing and eliminating effects of temporal X-ray/optical jitter, and characterizing and correcting for sources of various types of drifts that can lead to systematic errors in the measured data.

Some of these experimental tasks are relatively straightforward to solve (Minitti *et al.*, 2015 ▸). Spatial overlap between the X-ray and optical lasers is commonly achieved using Ce:YAG screens, knife-edge scans or even thermally activated liquid-crystal paper. As for temporal overlap, in some cases the exact pump–probe delay corresponding to the overlap of the X-ray and optical pulse, time zero (*T*_0_), can be directly measured from the sample response itself if the signal is large enough to be quickly measured. However, it is often necessary to use alternative methods that will be described in this article. Other aspects of the experiment such as timing drift correction, increasing scanning efficiency and correlation plot techniques will also be discussed.

## 2. Time zero determination   

### 2.1. Rough timing   

Rough timing down to the sub-10 ps level between the laser and X-rays is often achieved by inserting a device with a fast time response for both the X-rays and optical laser into the interaction region. The signal from the device is then measured on a remotely controlled oscilloscope, typically with a bandwidth exceeding 10 GHz and a sample rate of >20 Gigasamples per second. Metal–semiconductor–metal (MSM) detectors with a very broadband response to both optical and X-ray light and 30 ps response time (for example, Hamamatsu G4176) have been widely used in the LCLS X-ray hutches for rough timing (Chollet *et al.*, 2015 ▸; Alonso-Mori *et al.*, 2015 ▸). For hard X-ray experiments above 5 keV X-ray energy, we use the prompt X-ray fluorescence from a piece of titanium placed 45° from the MSM detector to record the arrival time of the X-ray pulse and enough optical light scatters from this target to also determine the optical laser timing. This method avoids the placement of the MSM detector directly at the sample location where it can be potentially damaged. In the case where the optical laser wavelength is out of the response range of the MSM detector, the center conductor of a sub-miniature version A connector wire or connector can also be used to provide a fast timing signal produced by light-field-induced charge carriers in the metal. In any method, a reference trace of the arrival time of the X-ray pulse on the detector is recorded on the oscilloscope and the optical laser delay is then adjusted to match the optical laser pulse arrival time with the X-ray pulse at the sample location.

It is also necessary to measure the rough timing between the X-ray and optical laser at the X-ray/optical cross correlator (Time Tool) location. From one experiment to another, depending on the optical laser source required, be it standard 800 nm, an optical parametric amplifier for visible light, or terahertz, the path length of the optical laser to the sample location can change by several meters relative to the Time Tool optical path. A similar setup using a titanium target and MSM detector is permanently affixed to the target paddle inside the Time Tool chamber to roughly co-time the X-ray with the optical laser at the Time Tool location. Once the rough timing has been determined both at the sample and Time Tool locations, we can ensure that the optical pump path length is within the mechanical delay line range used on the Time Tool path. If the path length difference is too big, we adjust the delay stage location on the Time Tool path and repeat the rough timing measurement.

### 2.2. Fine timing   

**Figure 1:**
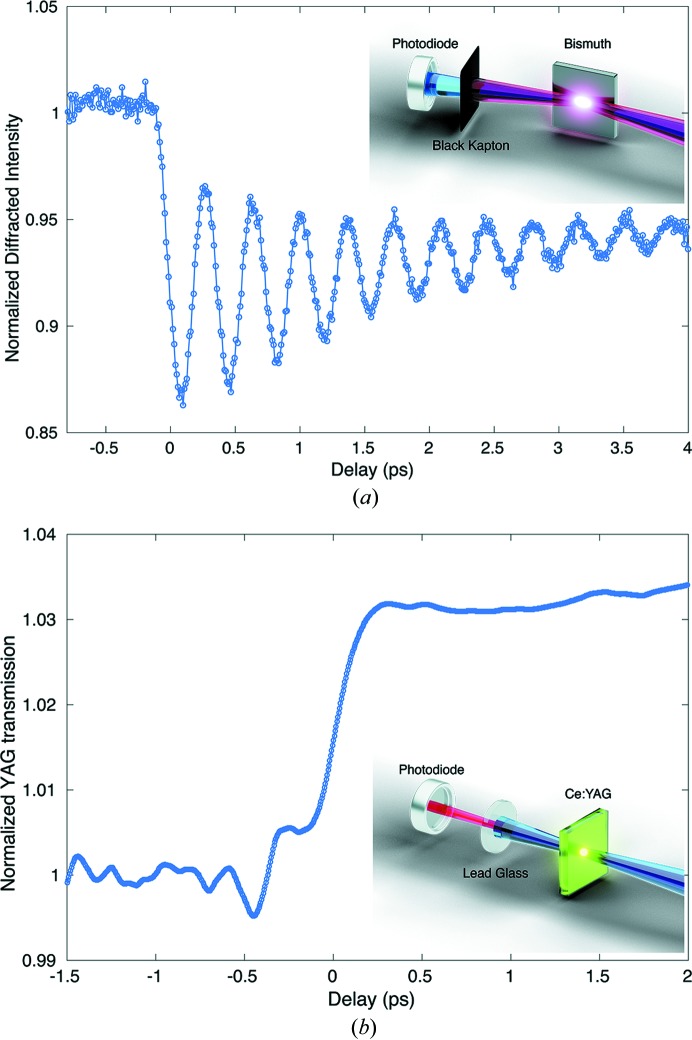
Typical time traces used to determine temporal overlap between the X-ray and optical laser and their respective schematic setup (X-ray in blue and optical laser in red). (*a*) Coherent phonon oscillations observed in bismuth (111) Bragg reflections. (*b*) Transmission change in Ce:YAG crystals from X-ray-generated free charge carriers.

More precise timing down to the ∼100 fs level can be found using a variety of techniques depending on the X-ray wavelengths and sample environment. In the case of an experiment performed in air with enough available space around the sample area, we often use the dynamic response of a bismuth thin film (50 nm) as a timing reference. Bismuth is a material that has very strong coupling between electronic and ionic structure, which makes it a good model system with extremely large phonon amplitudes. We align the (111) Bragg reflection to detect the coherent oscillations from laser-generated phonons. (Fritz *et al.*, 2007 ▸; Epp *et al.*, 2017 ▸). We use a 1 inch-diameter passivated implanted planar silicon (PIPS) diode to measure and center the (111) diffraction peak, and to avoid optical laser light contamination, the diode is shielded with black polyimide film or aluminium foil [Fig. 1 ▸(*a*), inset]. The optical laser delay is then stepped and the onset of coherent oscillations is indicative of the X-ray/optical laser overlap time [Fig. 1 ▸(*a*)]. The coherent oscillations shown were generated by a 40 fs full width at half-maximum (FWHM) 800 nm optical laser and 50 fs FWHM X-rays, and the blue line is a fit to the data as a visual guide. The generation of phonons simply requires optical absorption near the bismuth surface and it has been successfully used for laser wavelengths from the UV to the infrared. One disadvantage of this method is the relatively slow displacement of the atomic sites in the bismuth compared with the length of the X-ray and optical pulses. This causes the time resolution in finding *T*_0_ to be of the order of 100 fs FWHM, which is generally acceptable for most experimental measurements. The ultimate resolution of our pump–probe setup was recently bounded by K. Meckseki and collaborators (Mecseki *et al.*, 2018 ▸) in a study on ultrafast X-ray-induced electron-cascading processes in solids. A ∼25 fs FWHM hard X-ray pulse produced charge carriers in a thin sample of SnO_2_ that was probed by observing the transmission of an off-axis 9 fs FWHM 800 nm optical laser pulse, and carrier dynamics of the order of 30 fs FWHM were observed which were consistent with theory. Additionally, the experimentally derived arrival times of the optical and X-ray laser in this measurement correlated with the standard XPP Time Tool values to 5.2 fs RMS, which we believe is closer to the true limit of the time resolution at XPP when using 9 fs optical pump pulses.

For more space-constrained setups, where it is not easy or possible to measure a diffracted beam, we use a Ce:YAG crystal (Sanchez-Gonzalez *et al.*, 2017 ▸) in transmission geometry [Fig. 1 ▸(*b*), inset]. In this case, the X-ray pulses act as the pump and we detect the optical transmission change of the Ce:YAG caused by X-ray-produced free charge carriers in the Ce:YAG crystal [Fig. 1 ▸(*b*); Medvedev *et al.*, 2013 ▸]. We also often use a PIPS diode with this technique to measure the transmitted optical laser, but standard CCD cameras can also be used. When using the PIPS diode, the X-rays are blocked before the diode using a leaded glass window. The response lifetime from the YAG is of the order of microseconds, which makes it easy to initially detect the pump effect using a correlation plot technique which will be described in a later section (Sato *et al.*, 2019 ▸). Since both X-ray and optical laser light produce visible light in the Ce:YAG crystal, this setup is used to perform spatial overlap as well. The only real dis­advantage of a ‘thick’ (about 100 µm thick) YAG is the group velocity mismatch between the X-rays and the optical light, which produces a significant smearing of the edge feature around 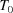 when the optical light is detected in transmission geometry. This can be improved by using a very thin crystal or by using imaging that is only sensitive to the surface (Sato *et al.*, 2019 ▸).

Once 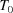 is determined at the sample location, same as with rough timing we proceed to find 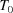 at the beamline Time Tool location upstream of the sample interaction region (Liang *et al.*, 2015 ▸; Chollet *et al.*, 2015 ▸; Alonso-Mori *et al.*, 2015 ▸). The Time Tool can use various targets depending on the X-ray intensity and wavelength from 1 µm- to 2 µm-thick Si
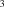
N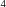 membranes as well as a 20 µm-thick Ce:YAG crystal. This measurement is destructive for the X-rays, and the transmission at 5 keV, 9 keV and 12 keV photon energies for 2 µm-thick Si_3_N_4_ is 90%, 98% and 99%, and for 20 µm-thick Ce:YAG is 9%, 65% and 82%, respectively. We generally use as thin a target as possible to maximize the flux going to the experiment, but if we are using a monochromatic beam, for instance, we must use a thicker target to obtain a resolvable signal. We have not noticed a significant difference in the timing resolution with respect to the target thickness for most experiments, but this may come into play on the sub-30 fs FWHM level where the group velocity mismatch in the YAG becomes significant. The ultimate resolution of the Time Tool is limited by a combination of many factors such as the shapes of the individual X-ray pulses, the uncertainty in the edge-finding algorithm, the different optical paths between the Time Tool and the interaction point, shot-to-shot fluctuations in the white light and/or noise floor, *etc*. Studies on schemes to increase the resolution are currently in development and we have been trying methods similar to those developed at the soft X-ray end-stations (Hartmann *et al.*, 2014 ▸).

Once 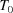 is determined at both the Time Tool and sample locations we can rely on solely monitoring the Time Tool signal to confirm that the temporal overlap at the sample location is maintained over the course of the experiment. Using our online monitoring tools we can quickly detect any large timing jump (more than 2 ps) and adjust the electronic laser delay to recover the Time Tool signal and be confident that the temporal overlap at the sample location was also recovered. For smaller timing jump or drift of the order of ∼100 fs, we rely on our drift monitor.

### 2.3. Timing drift monitor   

**Figure 2:**
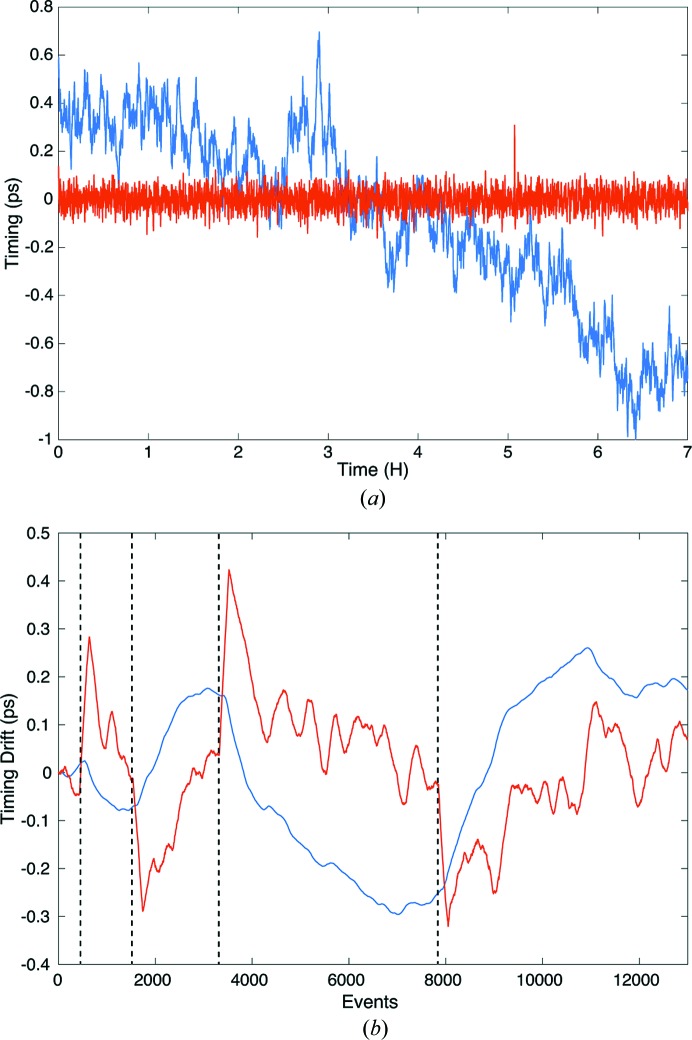
(*a*) Timing drift monitor over a 7 h time period. The Time Tool signal (red trace) stayed centered around time 0 and the blue trace shows the drift-correction value applied to the laser phase shifter in order to maintain time 0. (*b*) The Time Tool value is shown in red and the drift correction factor is shown in blue. The vertical dotted lines represent artificial time jumps of ±300 fs and ±400 fs to observe the response of the drift monitor feedback loop.

It is not uncommon for the X-ray/optical laser timing to slowly drift over a long period of time. For example, we measured around 1.5 ps drift over 7 h, as shown in Fig. 2 ▸(*a*). In the early days of pump–probe experiments at LCLS it was good practice to check spatial and temporal overlap at the beginning and periodically throughout a 12 h shift. For this reason, it became clear that being able to quickly monitor timing drift *in situ* was essential and would save valuable time during experiments.

In order to monitor timing drift, we take advantage of the Time Tool instrument. Time Tool is a relative time-of-arrival monitor that is commonly used to apply a temporal correction factor to each shot based on how far that particular shot was from the requested time delay. We can also use this value to monitor slow timing drift over time as long as the timing remains within the Time Tool window of ∼2 ps. To avoid over-correcting the timing drift, Time Tool values are filtered based on X-ray pulse energy and the Time Tool signal amplitude to only account for good X-ray and laser shots. These filtered Time Tool values are then used in a proportional integral derivative (PID) feedback loop and, if a temporal drift is detected, a correction factor is directly applied to the laser RF phase shifter system to maintain 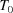 at its original position (Gumerlock *et al.*, 2014 ▸). Fig. 2 ▸(*b*) shows the recorded Time Tool values in red and the drift-monitor correction factor in blue. The vertical dotted lines represent instances where we artificially changed the timing by ±300 fs and ±400 fs, respectively, to observe the drift-monitor response. Using a combination of reference oscilloscope traces of the optical laser pulse timing for a large time window and the real-time Time Tool signal for a short time window, we can quickly detect any timing jump and correct it before continuing the experiment. A current drawback of the drift-monitor system is that it is only effective when our data acquisition system (DAQ) is actively running and Time Tool data are measured. When the DAQ is not running, the timing correction factor stops being updated and waits for new data.

### 2.4. Correlation plot techniques   

**Figure 3:**
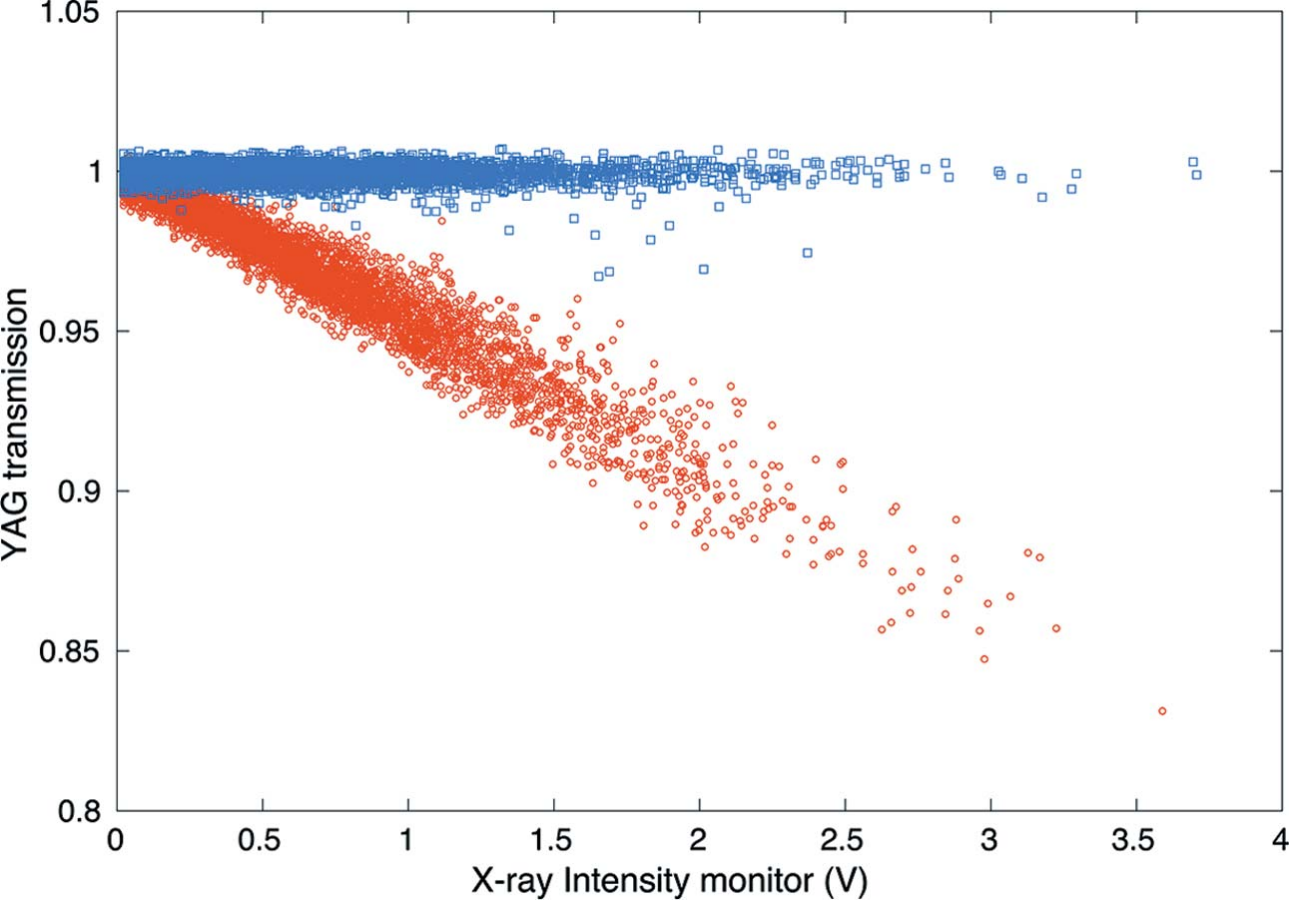
Correlation plot between the X-ray intensity monitor and the transmission intensity of a Ce:YAG crystal. Before time 0 (blue trace) the Ce:YAG transmission is independent of the X-ray intensity. After time 0, when the crystal is pumped by the X-ray, the transmission intensity is directly correlated with the X-ray intensity. Large signals observable in the correlation plots can also be used to optimized other experimental parameters such as spatial overlap between the X-ray and optical laser.

Now that we have established 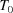 and are monitoring the timing for any jump or drift it is also important to pay attention to the noise level in the actual experiment. The SASE process induces pulse-to-pulse fluctuations of the beam properties, such as pulse energy, duration, spatial profile, wavefront, temporal profile and spectral content. This occurs even more so for experiments using the monochromatic beam because this often corresponds to roughly 100% intensity fluctuation in the transmitted beam. *In situ* pulse property monitoring is thus crucial for data interpretation. Multiple intensity position monitors (IPMs) (Feng *et al.*, 2011 ▸) are installed at various locations along the instrument for pulse-to-pulse intensity normalization. A correlation plot between the IPM signal and the signal from the sample side X-ray detector often quickly illustrates how noisy the pump–probe measurement will be. The better the correlation is, the lower the noise will be. It is therefore important to monitor this correlation and try to understand its source (*e.g.* sample grain size is too small compared with the X-ray focus, beam pointing instability for grazing incidence geometry, *etc*.) when trying to measure weak pump–probe signals of the order of or below 1% changes. On the other hand, when looking for large signals we can also directly use the correlation plot to detect and optimize the pump–probe signal. We commonly use this technique for liquid jet experiments where we use the correlation plots to optimize the jet position relative to the X-ray. If the X-ray is hitting the edge of the liquid jet this will result in a noisy correlation plot compared with when the X-rays are properly centered on the jet. We then strongly optically pump the liquid jet at large time delays (∼1 µs) where we know there is sensitivity to the solvent heating effect (Cammarata *et al.*, 2006 ▸). This signal is large and can easily be observed in the correlation plot when filtering between optical laser ON and optical laser OFF shots. Once we find the signal on the correlation plot, we can further optimize the spatial overlap between X-ray and optical laser by maximizing the difference between the two correlation traces. Another example where we rely on this correlation plot technique is when performing fine timing with the YAG screen method. As shown in Fig. 3 ▸, when the YAG screen is pumped by the X-ray, the optical laser transmission will start to correlate with the X-ray intensity. The change in the correlation slope is easy to detect and optimize.

To help further improve the signal-to-noise ratio, even with a drifting X-ray machine, we developed a novel laser timing scheme where laser pulses can be made to come after the X-rays on demand (often 0.1 µm to 50 µm). These pulses are effectively laser dark or X-ray only pulses that can be used as references to track and compensate for slow drifts to ‘flatten’ the baseline. This technique also has the crucial advantage that the sample, laser and laser-delivery optics all have consistent thermal loading by the laser, so the only consequence to the actual measurement is reduced statistics. The sequence and frequency of laser drop shots can be easily customized with an event sequencer which in return will send the ON or delayed (OFF) triggers to the laser system. Each shot has a tag in the datafile to easily identify whether it was a laser ON or OFF shot. When looking for very weak pump signal, the measurement noise can sometimes lead to the wrong assumptions about the signal fidelity and location. Being able to display both laser ON and laser OFF shots during a measurement is a great way to confirm that the weakly observed signals are indeed real time-resolved effects and not just random noise.

## 3. Time scan methods   

### 3.1. Scanning techniques   

Standard scanning techniques for pump–probe experiments rely on scanning either a delay stage or the RF phase of the laser-locking electronics. At LCLS, we use a combined approach of both techniques for most experiments. The RF phase shifter is used to linearly vary the timing during time scans and we use a mechanical delay stage to compensate the phase shifter timing change on the Time Tool optical path. The Time Tool timing needs to remain constant relative to the X-rays during time scans to provide us with the timing correction value for each shot. One of the major drawbacks of this standard scanning technique can be the overhead or dead time from our DAQ system. The time it takes to move the stages back and forth as well as the communication time between the stage controllers and DAQ can add significant overhead to a scan. The standard scanning method, scanning in one constant direction, can also suffer from potential X-ray beam dropouts, which can force us to redo a scan if the machine is too unstable for some time delays. There are also contributions to an uneven noise floor or signal strength from other sources of slow drift like beam pointing or slow sample damage. To help with the machine instability, we implemented a level 3 trigger which sets boundaries for good X-ray shots. If the machine performs poorly, the scan will pause and wait for recovery before resuming. This has greatly helped to reduce the amount of scan repetitions due to machine drop shots.

**Figure 4:**
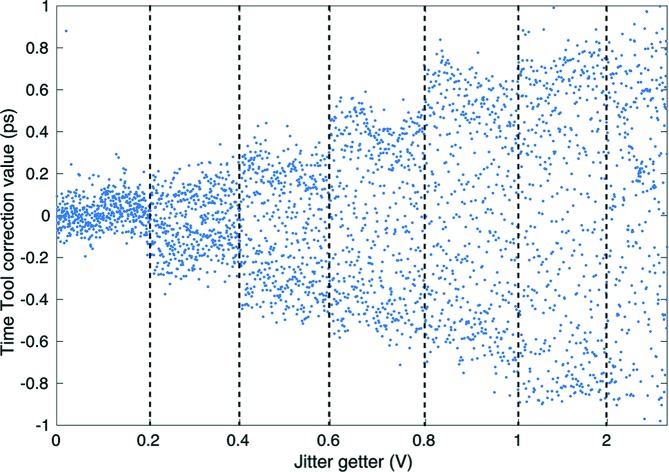
By manipulating the optical laser radio frequency locking system we can increase the timing jitter to randomly cover a larger time window from 200 fs to 2 ps.

**Figure 5:**
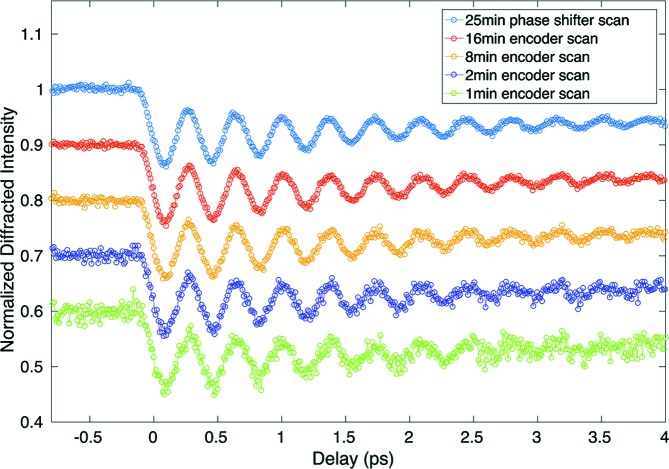
Comparison between a regular phase shifter time scan and the new encoder stage scanning technique. The encoder stage technique offers the advantage of revealing the complete time trace in a fraction of the time needed compared with the regular phase shifter scan. Weaker signal features require more statistics and the scan duration to extract the weaker features from the noise ends up comparable with the regular phase shifter scan.

To avoid overhead time from scanning the phase shifter and mechanical delay stage it was found that artificially increasing the timing jitter between the optical laser and X-rays through manipulating the RF locking system provides a convenient way to quickly scan up to a few picoseconds time window as shown in Fig. 4 ▸. In this scheme, an unsynchronized signal is introduced into the locking electronics, and the amplitude of this signal is roughly proportional to the observed optical laser timing jitter. This method is good for quickly exploring an experimental parameter space before sources of slow systematic drift become significant. Additionally, we have implemented an encoded fast-scan linear motor stage (Newport XMS-50) with a precision position encoder to expand this range up to 100 ps and to allow users to continuously and rapidly move timing back and forth through a given window without having any dead time. Fig. 5 ▸ shows a comparison between a regular 25 min-long phase shifter scan and encoder stage scans for various durations. The encoder stage scan yields the complete time trace in a fraction of the time needed compared with the regular phase shifter scan, where one needs to wait for the scan to be completed to observe the complete time trace. As shown in Fig. 5 ▸, a 1 min encoder scan is already sufficient to determine 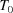 precisely but smaller oscillation features at later times are not yet well resolved. The features become more clear as the stage continues scanning for a few more minutes, and the counting statistics approach those of the 25 min phase shifter scan after about 8 min. One can also think of more unconventional scanning schemes where the encoder stage is continuously scanned back and forth over a given time window and the phase shifter timing is periodically changed to offset the scanning window. All this can be done within the same scan and can be easily sorted in the datafile.

## 4. Summary   

X-ray/optical pump–probe experiments are fairly well established at synchrotron sources nowadays; however, directly transporting these experimental techniques to the FEL sources is not always feasible considering the much shorter X-ray pulse duration and the intrinsic noise from SASE. It was necessary to develop new methods and tools to successfully perform these types of experiments at LCLS. In this article, we have presented the most commonly used techniques at LCLS to perform pump–probe experiments and the tools we used to streamline the setup and monitor key parameters during user experiments. These include from how to perform a rough temporal overlap with an MSM detector to fine timing using standard samples such as bismuth and Ce:YAG. Such measurements are possible thanks to the advancement in online monitoring tools and feedback methods to keep track of potential issues during an experiment.

## References

[bb1] Alonso-Mori, R., Caronna, C., Chollet, M., Curtis, R., Damiani, D. S., Defever, J., Feng, Y., Flath, D. L., Glownia, J. M., Lee, S., Lemke, H. T., Nelson, S., Bong, E., Sikorski, M., Song, S., Srinivasan, V., Stefanescu, D., Zhu, D. & Robert, A. (2015). *J. Synchrotron Rad.* **22**, 508–513. 10.1107/S1600577515004397 PMC4416668 25931061

[bb2] Bionta, M. R., Lemke, H. T., Cryan, J. P., Glownia, J. M., Bostedt, C., Cammarata, M., Castagna, J.-C., Ding, Y., Fritz, D. M., Fry, A. R., Krzywinski, J., Messerschmidt, M., Schorb, S., Swiggers, M. L. & Coffee, R. N. (2011). *Opt. Express*, **19**, 21855–21865. 10.1364/OE.19.021855 22109037

[bb3] Cammarata, M., Lorenc, M., Kim, T. K., Lee, J. H., Kong, Q. Y., Pontecorvo, E., Lo Russo, M., Schiró, G., Cupane, A., Wulff, M. & Ihee, H. (2006). *J. Chem. Phys.* **124**, 124504. 10.1063/1.2176617 16599694

[bb4] Chollet, M., Alonso-Mori, R., Cammarata, M., Damiani, D., Defever, J., Delor, J. T., Feng, Y., Glownia, J. M., Langton, J. B., Nelson, S., Ramsey, K., Robert, A., Sikorski, M., Song, S., Stefanescu, D., Srinivasan, V., Zhu, D., Lemke, H. T. & Fritz, D. M. (2015). *J. Synchrotron Rad.* **22**, 503–507. 10.1107/S1600577515005135 PMC4416667 25931060

[bb5] Daranciang, D., Highland, M. J., Wen, H., Young, S. M., Brandt, N. C., Hwang, H. Y., Vattilana, M., Nicoul, M., Quirin, F., Goodfellow, J., Qi, T., Grinberg, I., Fritz, D. M., Cammarata, M., Zhu, D., Lemke, H. T., Walko, D. A., Dufresne, E. M., Li, Y., Larsson, J., Reis, D. A., Sokolowski-Tinten, K., Nelson, K. A., Rappe, A. M., Fuoss, P. H., Stephenson, G. B. & Lindenberg, A. M. (2012). *Phys. Rev. Lett.* **108**, 087601. 10.1103/PhysRevLett.108.087601 22463572

[bb6] Epp, S. W., Hada, M., Zhong, Y., Kumagai, Y., Motomura, K., Mizote, S., Ono, T., Owada, S., Axford, D., Bakhtiarzadeh, S., Fukuzawa, H., Hayashi, Y., Katayama, T., Marx, A., Müller-Werkmeister, H. M., Owen, R. L., Sherrell, D. A., Tono, K., Ueda, K., Westermeier, F. & Miller, R. J. D. (2017). *Struct. Dyn.* **4**, 054308. 10.1063/1.4999701 PMC5658228 29152535

[bb7] Feng, Y., Feldkamp, J. M., Fritz, D. M., Cammarata, M., Robert, A., Caronna, C., Lemke, H. T., Zhu, D., Lee, S., Boutet, S., Garth, W., Tono, K., Yabashi, M. & Hastings, J. B. (2011). *Proc. SPIE*, **8140**, 8140OQ.

[bb8] Fritz, D. M., Reis, D. A., Adams, B., Akre, R. A., Arthur, J., Blome, C., Bucksbaum, P. H., Cavalieri, A. L., Engemann, S., Fahy, S., Falcone, R. W., Fuoss, P. H., Gaffney, K. J., George, M. J., Hajdu, J., Hertlein, M. P., Hillyard, P. B., Horn-von Hoegen, M., Kammler, M., Kaspar, J., Kienberger, R., Krejcik, P., Lee, S. H., Lindenberg, A. M., McFarland, B., Meyer, D., Montagne, T., Murray, E. D., Nelson, A. J., Nicoul, M., Pahl, R., Rudati, J., Schlarb, H., Siddons, D. P., Sokolowski-Tinten, K., Tschentscher, T., von der Linde, D. & Hastings, J. B. (2007). *Science*, **315**, 633–636. 10.1126/science.1135009 17272718

[bb9] Glownia, J. M., Cryan, J., Andreasson, J., Belkacem, A., Berrah, N., Blaga, C. I., Bostedt, C., Bozek, J., DiMauro, L. F., Fang, L., Frisch, J., Gessner, O., Gühr, M., Hajdu, J., Hertlein, M. P., Hoener, M., Huang, G., Kornilov, O., Marangos, J. P., March, A. M., McFarland, B. K., Merdji, H., Petrovic, V. S., Raman, C., Ray, D., Reis, D. A., Trigo, M., White, J. L., White, W., Wilcox, R., Young, L., Coffee, R. N. & Bucksbaum, P. H. (2010). *Opt. Express*, **18**, 17620–17630. 10.1364/OE.18.017620 20721148

[bb10] Gray, A. X., Hoffmann, M. C., Jeong, J., Aetukuri, N. P., Zhu, D., Hwang, H. Y., Brandt, N. C., Wen, H., Sternbach, A. J., Bonetti, S., Reid, A. H., Kukreja, R., Graves, C., Wang, T., Granitzka, P., Chen, Z., Higley, D. J., Chase, T., Jal, E., Abreu, E., Liu, M. K., Weng, T.-C., Sokaras, D., Nordlund, D., Chollet, M., Alonso-Mori, R., Lemke, H., Glownia, J. M., Trigo, M., Zhu, Y., Ohldag, H., Freeland, J. W., Samant, M. G., Berakdar, J., Averitt, R. D., Nelson, K. A., Parkin, S. S. P. & Dürr, H. A. (2018). *Phys. Rev. B*, **98**, 045104.

[bb11] Gumerlock, K., Frisch, J., Hill, B., May, J., Nelson, D. & Smith, S. (2014). *Proceedings of the 36th International Free Electron Laser Conference (FEL2014)*, 25–29 August 2014, Basel, Switzerland, pp. 917–921. THP080.

[bb12] Hartmann, N., Helml, W., Galler, A., Bionta, M., Grünert, J., Molodtsov, S., Ferguson, K., Schorb, S., Swiggers, M., Carron, S., Bostedt, C., Castagna, J.-C., Bozek, J., Glownia, J. M., Kane, D. J., Fry, A. R., White, W. E., Hauri, C. P., Feurer, T. & Coffee, R. N. (2014). *Nat. Photon.* **8**, 706–709.

[bb13] Lemke, H. T., Bressler, C., Chen, L. X., Fritz, D. M., Gaffney, K. J., Galler, A., Gawelda, W., Haldrup, K., Hartsock, R. W., Ihee, H., Kim, J., Kim, K. H., Lee, J. H., Nielsen, M. M., Stickrath, A. B., Zhang, W., Zhu, D. & Cammarata, M. (2013). *J. Phys. Chem. A*, **117**, 735–740. 10.1021/jp312559h 23281652

[bb14] Liang, M., Williams, G. J., Messerschmidt, M., Seibert, M. M., Montanez, P. A., Hayes, M., Milathianaki, D., Aquila, A., Hunter, M. S., Koglin, J. E., Schafer, D. W., Guillet, S., Busse, A., Bergan, R., Olson, W., Fox, K., Stewart, N., Curtis, R., Miahnahri, A. A. & Boutet, S. (2015). *J. Synchrotron Rad.* **22**, 514–519. 10.1107/S160057751500449X PMC4416669 25931062

[bb15] Mecseki, K., Höppner, H., Büscher, M., Tkachenko, V., Medvedev, N., Bekx, J. J., Lipp, V., Piekarz, P., Windeler, M., Tisch, J. W. G., Walke, D. J., Nakatsutsumi, M., Prandolini, M. J., Glownia, J. M., Sato, T., Sikorski, M., Chollet, M., Teubner, U., Robinson, J., Toleikis, S., Ziaja, B. & Tavella, F. (2018). *Appl. Phys. Lett.* **113**, 114102.

[bb16] Medvedev, N., Ziaja, B., Cammarata, M., Harmand, M. & Toleikis, S. (2013). *Contrib. Plasma Phys.* **53**, 347–354.

[bb17] Miller, N. A., Deb, A., Alonso-Mori, R., Glownia, J. M., Kiefer, L. M., Konar, A., Michocki, L. B., Sikorski, M., Sofferman, D. L., Song, S., Toda, M. J., Wiley, T. E., Zhu, D., Kozlowski, P. M., Kubarych, K. J., Penner-Hahn, J. E. & Sension, R. J. (2018). *J. Phys. Chem. A*, **122**, 4963–4971. 10.1021/acs.jpca.8b04223 29799204

[bb18] Minitti, M. P., Robinson, J. S., Coffee, R. N., Edstrom, S., Gilevich, S., Glownia, J. M., Granados, E., Hering, P., Hoffmann, M. C., Miahnahri, A., Milathianaki, D., Polzin, W., Ratner, D., Tavella, F., Vetter, S., Welch, M., White, W. E. & Fry, A. R. (2015). *J. Synchrotron Rad.* **22**, 526–531. 10.1107/S1600577515006244 PMC4416671 25931064

[bb19] Sanchez-Gonzalez, A., Johnson, A. S., Fitzpatrick, A., Hutchison, C. D. M., Fare, C., Cordon-Preciado, V., Dorlhiac, G., Ferreira, J. L., Morgan, R. M., Marangos, J. P., Owada, S., Nakane, T., Tanaka, R., Tono, K., Iwata, S. & van Thor, J. J. (2017). *J. Appl. Phys.* **122**, 203105.

[bb20] Sato, T., Glownia, J. M., Ware, M. R., Chollet, M., Nelson, S. & Zhu, D. (2019). *J. Synchrotron Rad.* **26**, 647–652. 10.1107/S1600577519002248 PMC6510204 31074427

[bb21] Schorb, S., Gorkhover, T., Cryan, J. P., Glownia, J. M., Bionta, M. R., Coffee, R. N., Erk, B., Boll, R., Schmidt, C., Rolles, D., Rudenko, A., Rouzee, A., Swiggers, M., Carron, S., Castagna, J.-C., Bozek, J. D., Messerschmidt, M., Schlotter, W. F. & Bostedt, C. (2012). *Appl. Phys. Lett.* **100**, 121107.

[bb22] Seddon, E. A., Clarke, J. A., Dunning, D. J., Masciovecchio, C., Milne, C. J., Parmigiani, F., Rugg, D., Spence, J. C. H., Thompson, N. R., Ueda, K., Vinko, S. M., Wark, J. S. & Wurth, W. (2017). *Rep. Prog. Phys.* **80**, 115901. 10.1088/1361-6633/aa7cca 29059048

[bb23] Teitelbaum, S. W., Henighan, T., Huang, Y., Liu, H., Jiang, M. P., Zhu, D., Chollet, M., Sato, T., Murray, E. D., Fahy, S., O’Mahony, S., Bailey, T. P., Uher, C., Trigo, M. & Reis, D. A. (2018). *Phys. Rev. Lett.* **121**, 125901. 10.1103/PhysRevLett.121.125901 30296113

[bb24] Wall, S., Yang, S., Vidas, L., Chollet, M., Glownia, J. M., Kozina, M., Katayama, T., Henighan, T., Jiang, M., Miller, T. A., Reis, D. A., Boatner, L. A., Delaire, O. & Trigo, M. (2018). *Science*, **362**, 572–576. 10.1126/science.aau3873 30385575

[bb25] Zerdane, S., Collet, E., Dong, X., Matar, S. F., Wang, H. F., Desplanches, C., Chastanet, G., Chollet, M., Glownia, J. M., Lemke, H. T., Lorenc, M. & Cammarata, M. (2018). *Chem. Eur. J.* **24**, 5064–5069. 10.1002/chem.201704746 29105179

